# Ultrafast plasmonic rotors for electron beams

**DOI:** 10.1515/nanoph-2025-0037

**Published:** 2025-05-22

**Authors:** Fatemeh Chahshouri, Nahid Talebi

**Affiliations:** Institute of Experimental and Applied Physics, Kiel University, 24098 Kiel, Germany; Kiel, Nano, Surface, and Interface Science – KiNSIS, Kiel University, 24098 Kiel, Germany

**Keywords:** plasmonic rotors, photon-induced near-field electron microscopy, electron wavepacket shaping, localized electromagnetic fields, angular momentum transfer

## Abstract

The interaction between free electrons and laser-induced near-fields provides a platform to study ultrafast processes and quantum phenomena while enabling precise manipulation of electron wavefunctions through linear and orbital momentum transfer. Here, by introducing phase offset between two orthogonally polarized laser pulses exciting a gold nanorod, we generate a rotating plasmonic near-field dipole with clockwise and counterclockwise circulating orientations and investigate its interaction with a slow electron beam. Our findings reveal that the circulation direction of plasmonic fields plays a crucial role in modulating electron dynamics, enhancing coupling strength, and controlling recoil. Furthermore, synchronizing the interaction time of the electron beam with rotational dipolar plasmonic resonances results in significant transfer of angular momentum to the electron beams and deflects the electron wavepackets from their original trajectory. These findings highlight the potential of plasmon rotors for shaping electron wavepackets, offering promising applications in ultrafast microscopy, spectroscopy, and quantum information processing.

## 1 Introduction

Recent years, innovations in electron microscopy have revolutionized nanoscience, enabling atomic-scale insights into biological, chemical, and semiconductor materials [1]. Moreover, the integration of coherent electron beams with femtosecond laser pulses [2] has further advanced electron microscopy, enabling the exploration of quantum phenomena [3], ultrafast charge oscillations [4], [5], and nonequilibrium optical excitations [6], [7]. Electron-Driven Photon Sources (EDPHS [8], [9], [10], [11]) within electron microscopes additionally have pushed the field further, facilitating interferometry and time-resolved spectroscopy with femtosecond time resolution [12] without the need for external lasers. Such developments have unlocked new possibilities for studying plasmon resonances [13], [14], [15], exciton dynamics [12], and phonon behavior [6], thereby driving breakthroughs in electron holography [16], [17], phase retrieval [18], attosecond pulse trains [19], [20], and wave packet shaping [13], [21], [22], [23].

Shaped electron wave functions have been shown to allow precise control over quantum electrodynamic interactions, scattering processes, and Bremsstrahlung emission [24]. Furthermore, shaped electron beams can enhance X-ray generation [25], and enable the distinction between different quantum interference pathways [26]. This approach also leads to advancements in imaging resolution [27], [28], selective probing [29], [30], low-dose imaging [31], quantum computing [32], and enhancing data transmission [33]. While traditional methods, such as nanofabricated phase masks [34], [35], [36], [37], magnetic field [38], and phase plate [39] can manipulate electron wavepackets, they are limited in terms of speed, active controlling, and concomitant transverse and longitudinal phase modulation of the electron wavepackets.

Ultrafast electron microscopy (UTEM), where electron wavepackets are used to probe laser-induced excitations in matter has in addition led to coherent and spatiotemporal shaping of electron wave functions [40], [41], [42]. In principle coherent light can be used for modulation [43], [44] of both longitudinal [13], [45], [46], [47] and transverse wave functions [48], [49]. These interactions occur either in free space, through coupling via the ponderomotive force [50], [51], [52], [53], [54], [55], [56] of a light wave, or within the optical near-fields of nanostructures excited by laser pulses [50], [57], where the latter is known as photon-induced near-field electron microscopy (PINEM) [4].

PINEM enables exploring the dynamics of near-field excitations by analyzing photon-electron longitudinal momentum exchange versus the delay between the electron wavepackt and light pulses [2]. In such interactions, the coupling strength [4], which governs energy exchange with electrons, can be enhanced by reducing mode volume, employing dielectric medium, increasing the longitudinal electric field, or extending interaction lengths [31]. Therefore, extended mode lifetimes to the picosecond range in systems like photonic crystals, or whispering gallery modes [14], [58] results in more quanta of energy exchange between laser and electron wavepackets, i.e., more PINEM peaks. However, resonant phase-matching, achieved by matching electron velocity with phase velocity of light in a prism [59] has also demonstrated the exchange of hundreds of photon quanta with single electrons over long distances. Slow electrons interacting with localized plasmonic fields [60], [61] can also enhance the coupling coefficient by increasing the effective interaction time [13], [62]. Furthermore, it has been shown that, beyond the near-field-mediated regime, the vector potential of freely propagating light waves in systems utilizing a single Hermite–Gaussian laser pulse [63], stimulated Compton scattering [20], and optical beat waves [64] can also result in inelastic scattering of electron beams.

The electron temporal coherence relative to the light period determines the interaction regime for the modulation of the electron beam. With few-cycle THz pulses [65], microwaves [66] or radio waves [67] where temporal coherence of the electron wave packet is shorter than the light period $\left(\Delta t_{e}<t_{\mathrm{ph}}\right)$ the electron spatial distribution follows classical electron deflection. In contrast, for an electron temporal coherence longer than the light period for example in near-infrared light [40] interactions gives rise to quantum dynamics, greatly influenced by the electromagnetic vector potential. In this regime, the wave nature of the electron becomes pronounced, enabling coherent quantum effects such as diffraction. A prominent example of such quantum interaction is the Kapitza–Dirac effect, where an electron wavepacket experiences diffraction when passing through periodic gratings formed by counter-propagating laser beams [68]. This phenomenon arises from the ponderomotive potential and leads to the formation of transverse momentum sidebands spaced by 2*k*_ph_ [51], [52]. Furthermore, when the laser beams are instead incident obliquely [68], the resulting transverse diffraction becomes more complex due to quantum interferences between sequential single-photon processes and direct two-photon processes. Similarly, plasmonic Fabry–Perot cavities, formed by counter-propagating surface plasmon polaritons, can induce diffraction in the electron wave function [69]. Additionally, the Lorentz force generated by localized plasmons in gold nanorods [13], [21], [22], [62], acts as both a phase and amplitude grating, enabling elastic diffraction and inelastic energy transfer. Exciting the nanorods with linearly polarized light allows for the manipulation of the linear momentum of the electron wavepacket, whereas using circularly polarized light enables the transfer of angular momentum to the electron as well [43]. It has been demonstrated that precise phase modulation can be achieved by controlling nanostructure configurations [22], topology [13], and size [13], along with the spatial profile of near-fields [21], [22].

In this work, we introduce plasmonic rotors as a novel platform for manipulating free-electron wave functions. Here, we investigate the interaction of a slow electron beam with plasmonic rotors and examine how the direction of circulating dipolar plasmons controls the longitudinal and transverse recoil of the electron wavepacket. The plasmonic rotors are generated by two orthogonal laser pulses with perpendicular polarizations and a $\left(\pm \frac{\pi }{2}\right)$ phase offset, interacting with a gold nanorod. These rotors enable coherent and enhanced momentum transfer to electron wave packets by enhancing the interaction time and influencing the effective light frequency at the rest frame of the electron. By synchronizing the electron propagation with the plasmonic rotor, we demonstrate that its angular momentum and probability amplitude in both real and reciprocal space are significantly influenced by the direction of plasmon circulation. We further demonstrate that in clockwise (CW) rotation, where the electron propagation direction aligns with the near-field oscillation, the coupling strength and consequently the momentum transfer is enhanced. In contrast, in counterclockwise (CCW) rotation, the coupling strength decreases. This approach enables an additional degree of control over the ultrafast modulation of electron wave functions for transverse momentum as well as energy transfer, with applications in electron imaging [2], diffraction [70], [71], and spectroscopy [4]. Where, shaped electron beams, such as electron vortex beams [30], have potential for enhancing electron microscopy, particularly in the study of magnetic and biological specimens [72].

## 2 Materials and methods

To investigate the interaction between a laser-induced near-field and a free electron wavepacket beyond the adiabatic approximation [73], we have developed a self-consistent numerical framework that simultaneously solves Maxwell and Schrödinger equations [50]. For simplicity, we consider here the electron-light interaction in a two-dimensional space (*xy*-plane), with the electron propagating along the *x*-direction. Therefore, we study the near-field effects at the cross-section of a nanorod confined to the *xy*-plane, assuming the nanorod has infinite height along the *z*-axis. Since the angle of incidence and the polarization of the light are both in the *xy*-plane, the field is uniform along the *z*-axis and therefore, the electron wavepacket dynamics can be studied in the *xy*-plane. Consequently, the system can be effectively described using a two-dimensional Schrödinger equation without loss of generality. This assumption allowed us to focus on the laser-induced near-field at the cross section of a long rod with a high aspect ratio, thereby minimizing the influence of substrate and edge effects, this configuration have been experimentally demonstrated and used in the field of dielectric laser acceleration [74]. The plasmonic near-field properties in this framework are computed at each time step using the finite-difference time-domain (FDTD) method, where the gold permittivity is modeled using a Drude model with two critical point functions [50]. Subsequently, the field components are interpolated from the Maxwell domain into the Schrödinger frame. The time evolution of the electron wavepacket, $\left(\psi \left(\vec{r},t\right)\right)$ , in the vicinity of the laser-induced near-field is determined by solving the Schrödinger equation with the minimal-coupling Hamiltonian. After the interaction in the Schrödinger frame, the final electron wavepacket is analyzed to extract information on energy modulation and electron recoil. Finally, the inelastic scattering cross-section map is calculated using the final electron wavefunction $\left(\psi_{f}\left(x,y,t\to \infty \right)\right)$ , as [13]:

(1) $$\begin{array}{ll} \sigma \left(E,\varphi \right) & =\frac{d}{dEd\varphi }\langle \psi_{f}\left(x,y,t\to \infty \right)\left|\hat{H}\right|\psi_{f}\left(x,y,t\to \infty \right)\rangle \\ & =\left(\frac{m_{0}E}{\hbar^{2}}\right){\left|\tilde{\psi }\left(E,\varphi ;t\to \infty \right)\right|}^{2}. \end{array}$$

Here, *m*_0_ represents the electron mass, and *ℏ* denotes the reduced Planck constant. The electron kinetic energy is defined as $E=\hbar^{2}\left(k_{x}^{2}+k_{y}^{2}\right)/2m_{0}$ , and the scattering angle is given by $\varphi =\tan^{-1}\left(\frac{k_{y}}{k_{x}}\right)$ . $\tilde{\psi }$ is the wavepacket in the momentum space. Moving beyond the non-recoil approximation provides a more detailed perspective on the interaction, as it captures not only the longitudinal momentum distribution but also the amplitude modulation of the electron beam and its transverse momentum spread [13]. The transverse field component, on the other hand, induces lateral diffraction on the electron beam. The arrangement of diffraction orders at different energies is influenced by the electron velocity, the optical near-field momentum distributions, and the nanoparticle topology [13].

The electric field component of the near-field is the mediator for preserving the energy-momentum conservation in the system [2]. Therefore, when an electron with an initial momentum *p*_*e*_ = *ℏk*_*e*_ interacts with a laser-induced near-field, it absorbs or emits *n* quanta of photons from longitudinal component of the scattered filed. Consequently, its wavefunction evolves into a superposition of momentum states given by $p_{e}=\hbar \left(k_{e}+n\left(\omega_{\mathrm{ph}}/v_{e}\right)\right)$ . This process forms an energy comb, where the spacing between the peaks is determined by the photon energy *ℏω*_ph_. The probability amplitude $\left({\left|\psi_{n}\left(x,t\right)\right|}^{2}\right)$ for the exchange of *n* quanta of energy between the electron wavepacket and the near-field light is obtained by expanding the wavefunction as a Bessel series using the Jacobi–Anger relation:

(2) $${\left|\psi_{n}\left(x,t\right)\right|}^{2}\propto J_{n}^{2}\left(\left|g\right|\right).$$

Here *J*_*n*_ is the *n*th Bessel function of the first kind, and *g* represents the coupling strength of the electron-light interaction [4]:

(3) $$\begin{array}{ll} g & =\left(e/\hbar \omega_{\mathrm{ph}}\right)\int_{-\infty }^{\infty }dx^{\prime }{\tilde{E}}_{x}\left(x^{\prime },y;\omega_{\mathrm{ph}}\right)e^{-ix^{\prime }\omega_{\mathrm{ph}}/v_{e}}\\ & =\left(e/\hbar \omega_{\mathrm{ph}}\right){\tilde{E}}_{x}\left(k_{x}=\omega_{\mathrm{ph}}/v_{e},y;\omega_{\mathrm{ph}}\right). \end{array}$$

In the weak interaction regime, where *eg*/*ℏω*_ph_ ≪ 1, electron-light interactions give rise to a frequency comb of photon absorption and emission peaks when higher-order excitations exhibiting lower intensities than zero-loss peak [4]. In contrast, within the strong interaction regime a significant modification of the electron wave packet due to the coherent energy exchange with the optical field leads to an enhanced coupling and depletion of the zero-loss peak, and the emergence of higher-order elastic and inelastic processes, ultimately enabling electron-photon entanglement [75].

## 3 Results and discussion

In this work, we investigate the influence of circular plasmon resonances on the modulation of slow electron wavepackets. We demonstrate that applying two orthogonal laser pulses with a $\pm \frac{\pi }{2}$ phase offset introduces a dynamic phase relationship between the *x* and *y*-polarized near-field dipoles. This phase difference generates time-dependent dipole moments within the nanorod, which are not merely linear oscillations but instead exhibit rotational behavior driven by the evolving temporal phase difference. As a result, when the scattered wave includes a rotating dipole aligned with the electron motion, effective coupling with the electron wavefunction is achieved. Conversely, the dipole oscillates too fast in the rest frame of the electron and fails to facilitate effective coupling.

We model the interaction of a Gaussian electron wavepacket with a kinetic energy of 1 keV, a longitudinal broadening of *W*_L_ = 132 nm, and a transverse broadening of *W*_T_ = 15 nm with two laser pulses at a central wavelength of *λ* = 800 nm and a temporal broadening of 21 fs (all values throughout the manuscript are full-wave half maximum).

**Figure 1:**
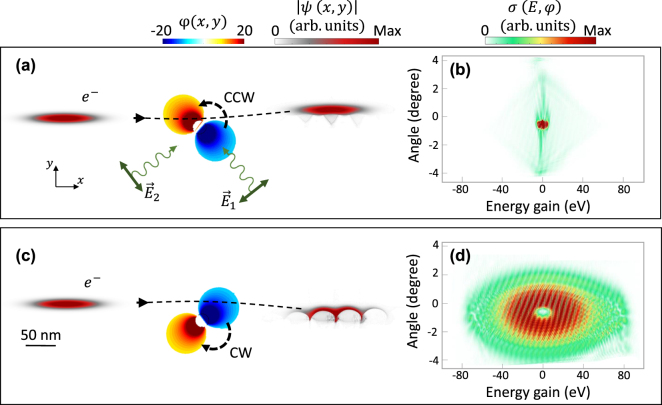
Electron beam shaping by a rotating localized plasmonic dipole. The localized plasmon resonance is generated by two orthogonally polarized laser pulses, with a $\pm \frac{\pi }{2}$ phase offset between them, generating a CCW or CW plasmonic rotor depending on the phase offset. The angle-resolved inelastic scattering cross section of the electron wavepacket after the interaction with the (a, b) CCW and (c, d) CW dipolar modes of a gold nanorod. Panels (a, c) depict the modulation of the amplitude of the electron wavepacket in real space before and after interaction with the rotational near-field modes, while (b, d) illustrate the inelastic scattering cross-section following the interaction. The phase and direction of the optical near-field are represented by $\text{Re}\left\{\phi \left(x,y\right)\right\}$ and black curved arrow, where $\phi \left(x,y\right)$ denotes the scalar potential. The electron beam has an initial centre kinetic energy of 1,000 eV, with longitudinal and transverse broadenings of 132 nm and 15 nm FWHM, respectively. The laser pulses feature a central wavelength of 800 nm, and FWHM temporal broadening of 21 fs, and a peak field amplitude of 2 GVm^−1^. Dashed arrows indicate the trajectory of the electron along the *x* direction. The gold nanorod has a radius of 25 nm.

These initial parameters are selected to satisfy the synchronicity condition [13], *λ*_ph_ *v*_e_ = 2*R* between the electron wavepacket propagation and effective dipolar mode oscillation, whereas keeping the interactions within the quantum regime, so that the longitudinal broadening is effectively longer than extend of the near-field. Here, *λ*_ph_ is the wavelength of the plasmonic resonances, *v*_e_ is electron velocity, and *R* is the radius of gold nanorod (*R* = 25 nm). Figure 1 illustrates the electron modulation in both real and momentum space after interaction with CCW (a, b) and CW (c, d) rotating near-field oscillations. The schematics on the left side of Figure 1 illustrate the amplitude modulation of the electron wavepacket in real space before and after interaction with the rotational near-field modes. The spatial distribution and the rotational direction of the optical near-field are represented by the scalar potential $\varphi \left(x,y\right)$ and the black curved arrow, respectively. As shown in Figure 1(a), CCW rotational field pushes the electron upward, increasing the impact parameter and reducing the degree of attosecond bunching in the electron wavepacket. Conversely, CW dipolar oscillations push the electron downward (Figure 1(c)), decreasing the impact parameter (illustrated in Supplementary Figure 1). To further quantify these effects, we analyze the average electron position after interaction with CW, CCW, *x*-, and *y*-polarized near-fields (Supplementary Figure 1). The results confirm a clear upward deflection in the CCW case and a downward shift in the CW case, in comparison to the single *x*- and *y*-polarized excitation schemes for the same initial phase setting. Additionally, the temporal oscillation of the laser pulses, influenced by their phase offset, introduces a ripple in the electron’s *y*-position, more pronounced for *y*-polarized light.

For the case of CW, the attractive force exerted on the electron wavepacket deflects the electron towards the nanorod, further leading to an enhancement of the interaction strength. Therefore, the ultrafast deflection experienced by the electron wavepacket is a factor affecting the strength of the interaction. In addition, the oscillation time of the projected field along the electron propagation direction plays a crucial role in shaping the electron beam. The Lorentz force exerted by the near-field induces a wiggling motion in the electron wavepacket that further controls the interaction. The direction and dynamics of this wiggling motion, plays a prominent role in the interaction strength and final extend of the wavepacket in the momentum space (see Supplementary Movie S1). For the case of CW rotations, this force acts synchronously with the electron motion and leads to a unified transverse recoil across the energy distribution. Therefore, the rotational direction of wiggling motion controls the final electron modulation in momentum representation. In contrast, contrary-aligned field rotation relative to the electron propagation direction has a destructive effect. As a result, in the CCW near-field case (Figure 1(b)), momentum transfer spans diffraction angles up to *φ* ≈ ± 3° within a small energy range of −5 eV ≤ *E* ≤ 5 eV. In comparison, the CW near-field (Figure 1(d)) induces stronger interactions. Consequently, the electron wavepacket experiences a transverse recoil spanning both positive and negative diffraction angles (−4° ≤ *φ* ≤ +4°), along with a broad longitudinal inelastic energy exchange within the range of −80 eV ≤ *E* ≤ 80 eV.

It should be noted that illuminating the nanorod with circularly polarized light propagating along the symmetry axis of the nanorod (the *z*-axis in our study) is another possible approach for exciting a rotating plasmonic dipole. However, as demonstrated in Supplementary Figure 2, this configuration breaks the two-dimensional symmetry of the excitation scheme, causing the electron wavepacket to experience and additional recoil along the *z*-axis.

**Figure 2:**
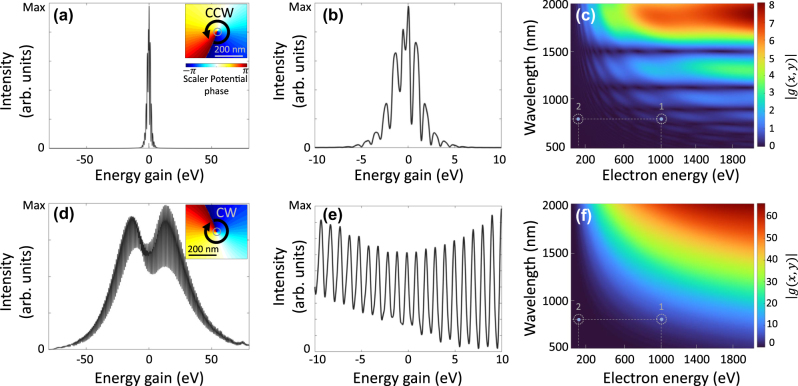
Impact of the near-field rotational direction on the energy transfer between light and free electron. PINEM spectra at for an electron with the initial energy of 1 keV are shown after interaction with a (a, b) CCW and (d, e) CW rotational localized plasmonic dipoles. Panels (b) and (e) highlight the PINEM spectra in a narrow range near the zero-loss peak. The insets in (a) and (d) illustrate the simulated phase maps of the rotational scalar potential, derived from FDTD calculations. Panels (c) and (f) present the calculated coupling parameter *g* at the center of the electron beam for CCW and CW field oscillations, respectively.

Figure 2(a), (b), (d), (e) better illustrates the role of the rotational direction of near-field in controlling the energy exchange between light and free electrons of the mentioned system. As demonstrated in insets of Figure 2(a) and, (d) via the phase of the scalar potential, these circularly CCW and CW near-fields create a singularity at the center of the nanorod. These figures also present the PINEM spectra of the electron under time-varying near-fields, when several energy peaks are observed. Examining the energy spectrum near the zero-loss peak reveals distinct differences between the CCW (Figure 2(b)) and CW (Figure 2(e)) configurations. In the CCW configuration, the small energy broadening $\left(\pm 5\text{ eV}\right)\;$ and irregular spacing between maxima indicate low coupling efficiency. Conversely, in the CW system, precise phase-matching produces a fine spectral structure with well-defined spacing between each photon order. Difference arises from the longitudinal component of the plasmonic field, which is primarily responsible for energy transfer. In the clockwise near-field configuration, dipolar field rotating along the electron beam propagation direction significantly amplifies the intensity of the PINEM energy spectra on both the gain and loss sides. In contrast, the counterclockwise design demonstrates the opposite effect, with reduced interaction strength and lower spectral intensity.

Studies of electron modulation with a single laser pulse further reveal that momentum transfer in the CW configuration exceeds that of purely *x*- or *y*-polarized light (Supplementary Figure S3). This enhanced transfer results from the combined action of *x*- and *y*-polarized dipolar plasmons that co-propagate with the electron. In contrast, for CCW field, the rotational restoring force against the electron propagation direction cancels out momentum exchange. Additionally, simulations using a broader electron wavepacket show similar sensitivity to the near-field, confirming the robustness of the effect (Supplementary Figure S4).

To provide a better understanding of the polarization-dependent interaction strength, we quantify the coupling strength using the *g*-factor, which characterizes the interaction efficiency between the near-field and the electron beam. Our semi-analytical approach, based on Hergert’s method for solving the time-dependent Schrödinger equation, confirms the numerical findings (see Supplementary Figure S5), revealing a strong asymmetry in the interaction strength for clockwise (CW) and counterclockwise (CCW) rotations. Although this approximation offers a simplified approach for modeling the system, it does not fully capture the complexities of electron recoil and its control, as it neglects vector-potential contributions and ponderomotive interactions.

The arrival time of the electron at the near-field does not play a significant role in the observed chiral-dependent interaction strength (Figure S6). This is due to the fact that a time-dependent shift in the electric field translates to a phase in the *g*-factor, but not altering its amplitude. By calculating a map of the coupling coefficient *g* (Eq. (3)) versus electron energies ranging from 20 to 2,000 eV and photon wavelengths between 500 and 2,000 nm, we observed the transition from the weak to strong coupling regime for rotational near-fields (see Figure 2(c) and (f)). These maps illustrate how the interaction strength varies with the electron energy and the wavelength of the incident light providing valuable insight into the dependence of the phase-matching criterium on the near-field properties. For instance, a slow electron beam with a kinetic energy of 100 eV interacting with a near-field excited by an 800 nm laser wavelength (illustrated by point 2 in Figure 2(c) and (f)) lies in a weak interaction regime. In this case, the phase-matching condition for energy and momentum transfer cannot be achieved (see Supplementary Figure 7). Comparing the *g*-coefficient highlights how the rotation of the near-field breaks symmetry in the phase-matching criteria and enables selective energy transfer in either the gain or loss channels. In the counterclockwise (Figure 2(c)) system, the intensity of *g* at the selected point (corresponding to the conditions in our simulation) is lower than in the clockwise (Figure 2(f)) configuration.

We find *g* = 4.38 for CW and *g* = 0.18 for CCW excitation, yielding a ratio of 24.3 between the two configurations. This significant difference arises from the alignment of the rotating direction of the near-field dipole with the electron’s motion. From a classical perspective, this effect translates to an effectively attractive force exerted on the electron in the CW case. Moreover, within the electron’s rest frame, a CW rotation leads to a decrease in the in the experienced photon frequency by the electron beam at the electron rest frame, due to the Doppler effect, that further enhances the coupling strength, while the CCW case results in a repelling force and effectively higher photon frequency. Then, the high coupling efficiency in the CW case is attributed to the unidirectional propagation of the plasmonic modes at the nanorod cross-section with the electron, ensuring relative motion that favors interaction with the rotating near-field. In contrast, from the electron rest frame, the counterclockwise system introduces a unidirectional propagating field that prevents energy-momentum matching. Consequently, a dark line appears in the map, marking regions of suppressed interaction. However, for single *x*- or *y*- polarized laser-induced near-fields, the coupling strength is lower than that of the CW configuration but higher than that of the CCW configuration (see Supplementary Figure 3).

More intriguing electron manipulation occurs when a point-projected electron beam passes through the near-field region. In this scenario, we analyzed a near-field area under the same initial conditions and electron energy as the previous system (Figures 1 and 2). However, the full-width at half-maximum (FWHM) of the longitudinal and transverse broadenings of the electron wavepacket are set to 15 nm and 132 nm, respectively.

**Figure 3:**
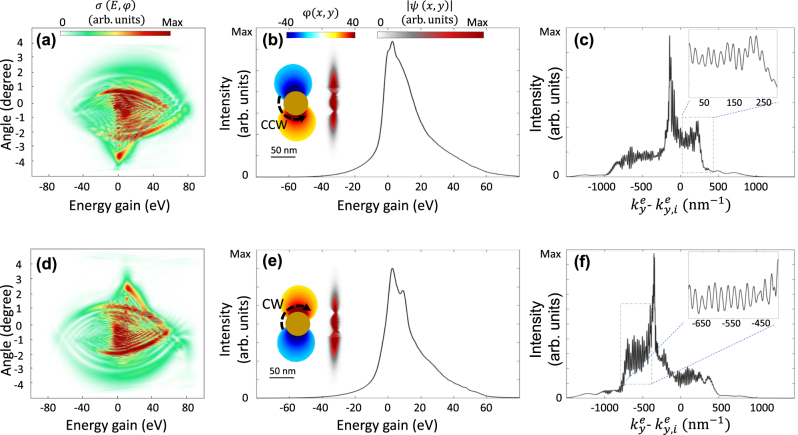
Deflection of a diverged electron beam influenced by near-field oscillation. Electron modulation spectra after interaction with (a–c) right-handed and (d–f) left-handed rotating plasmons. (a, d) Inelastic scattering cross-section of the electron wavepacket after the interaction with the near-field. (b, e) PINEM spectrum, real-space distribution of the electron wavepacket, and a snapshot of the induced plasmonic near-field circulation orientation. (c, f) Transverse recoil of the electron beam integrated over the full energy range, with insets showing the magnified spectrum within a selected range. The electron beam is characterized by a kinetic energy of 1,000 eV, with FWHM longitudinal and transverse broadenings of 15 nm and 132 nm. Where the laser pulses have a central wavelength of 800 nm, and a peak field amplitude of 2 GVm^−1^.

Figure 3 illustrates the modulation of the diverging electron wavepacket under the influence of counterclockwise rotating (CCW) (Figure 3(a)–(c)) and clockwise rotating (CW) field (Figure 3(d)–(f)) configurations. Within this framework, when the electron wavepacket enters the near-field region, its upper and lower parts experience a time-varying plasmonic field with a π phase difference. This phenomenon is analogous to the Aharonov–Bohm effect; however, in this case, the scalar potential term of the Hamiltonian governs the interaction (assuming Coulomb gauge). The opposing sides of the electron wavepacket interact with near-field potentials of opposite signs, causing the wavepacket to split into two distinct paths. Finally, the interference between these two parts generates unique interference and diffraction patterns on the detector, as reflected in the final inelastic scattering cross-section map. As the diverging electron wavepacket passes through the center of the nanorod region, it undergoes four complete oscillations (the dynamics of this electron modulation are illustrated in Supplementary Figures 8 and 9). The overall phase accumulated by the electron over multiple light-field cycles, combined with the direction of field oscillation, determines the final momentum span of the wavepacket in both transverse and longitudinal directions. Consequently, the opposite oscillation directions in the clockwise and counterclockwise configurations result in vertically flipped momentum modulation maps (Figure 3(a) and (d)). Whereas for the sake of a focused electron beam interacting with the rotating dipole, the interaction strength is significantly controlled by the direction of the rotation, for a diverging electron wavepacket, the interaction strength remains the same. However, a significantly asymmetric PINEM spectrum is observed for the latter case, that allows for selectively populating electron energy loss or gain channels.

The asymmetric force exerted by the oscillating fields causes significant transverse electron deflection after interaction with the near-field. The electron is deflected upward in the CCW configuration and downward in the CW configuration (see insets of Figure 3(b) and (e), respectively). Moreover, since the electron stays in the interaction region for a short time and its longitudinal broadening is small, the inelastic momentum exchange is weak. Consequently, the final PINEM spectra for both configurations exhibit a broadband spectral feature, as shown in Figure 3(b) and (e). Along the transverse direction, the electron wavepacket experiences a significant Kapitza–Dirac-like diffraction as well. This near-field-mediated diffraction produces significant angular deflections in the transverse direction, surpassing those observed in the free-space Kapitza–Dirac effect $\left(2k_{\text{ph}}\right)$ , where inverse spectra for CCW (Figure 3(c)), and CW (Figure 3(f)) near-fields are observed. Quasistatic approximations have been used elsewhere, when slow-electrons are used [62]. As illustrated in Supplementary Figure 10, this method captures part of the main features described by the full Hamiltonian system. The calculated coupling coefficient map reveals that electron-near-field interactions are significantly enhanced at higher electron velocities and lower near-field rotational speeds.

The latter is evident from eq. (3), since the *g*-factor is inversely proportional to the light frequency, while the former is captured by the electron-photon interaction selection rule $\left(k_{x}=\omega_{\mathrm{ph}}/v_{e}\right)$ . When phase-matching conditions between the electron and photons are satisfied, the electron recoil can be precisely manipulated, leading to strong modulation of the electron wavepacket. This enhancement can be achieved by increasing both the nanorod radius and the wavelength of the incident light. Higher electron velocities and reduced near-field frequencies extend the effective interaction time between the copropagating field and the electron, enabling efficient electron-near-field coupling. Such coupling, critical for observing higher photon orders, is achieved under carefully optimized conditions. To investigate this phenomenon, we analyzed the influence of optical near-fields near a gold nanorod with a radius of 80 nm. The electron wavepacket is characterized by an initial energy of 1,650 eV, with transverse and longitudinal broadenings of 25 nm and 320 nm, respectively. The incident laser wavelength is set at 2000 nm, and initial conditions is designed to ensure synchronicity between the electron and dipolar oscillations in both time-varying and static localized near-fields.

**Figure 4:**
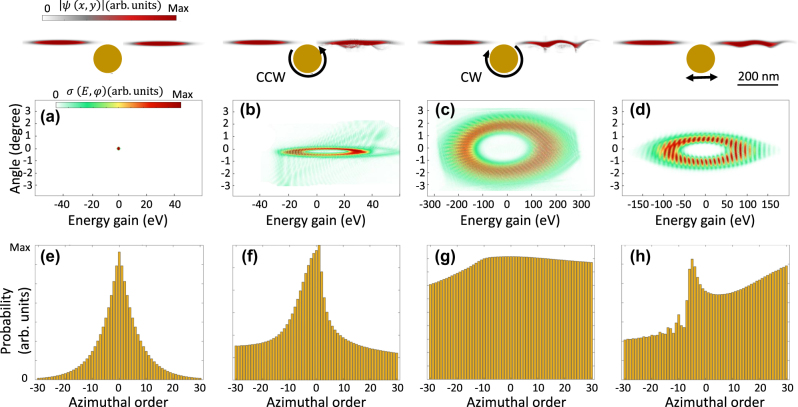
Probability amplitude distribution of the angular momentum transferred to the electron wavefunction by plasmonic near-field rotors. A Gaussian electron wavepacket, with a kinetic energy of 1,650 eV and transverse and longitudinal broadenings of 25 nm and 320 nm at FWHM, interacts with a plasmon generated by a nanorod with the radius of 80 nm. The inelastic scattering cross-section of the electron wavepacket is shown after propagation in (a) free space and interaction with (b) CCW, (c) CW, and (d) *X*-polarized plasmonic rotors. The upper row depicts the bunched electron profile after passing through the plasmonic near-field. Probability distribution of the angular momentum of the final electron wavefunction after propagating through (a) free space (no interaction), (b) CCW, (c) CW, and (d) *x*-oriented localized plasmonic dipolar fields. The laser pulse features a central wavelength of 2000 nm, an electric field amplitude of *E*_0_ = 1 GVm^−1^, and a temporal FWHM broadening of 53 fs, respectively.

Figure 4 illustrates four configurations of the interaction: (a) no near-field, (b) CCW near-field, (c) CW near-field, and (d) *x*-polarized near-field, highlighting their respective influences on the propagating electron wavepacket. After the interaction the electron beam amplitude bunches and its linear and angular momentum deviate from the characteristics of a simple Gaussian beam (Figure 4(a)). By carefully analyzing the angular momentum probability distribution, we observed that this intense interaction effectively imparts angular momentum to the electron wavepacket.

The inelastic scattering cross-section map reveals distinct differences in energy gain/loss and angular distributions between CCW (Figure 4(b)), CW (Figure 4(c)), and *x*-polarized (Figure 4(d)) near-fields. For all interaction types, the free-electron wavepacket experiences a strong interaction regime, characterized by depletion of the ground state (zero-line peak) in the final modulation map. However, due to the horizontal broadening of the electron wavepacket, the influence of the *x*-polarized near-field is significant. For the *x*-polarized system, we observed a substantial energy transfer to the electron, spanning from −150 eV to 150 eV. Adjusting the phase matching by changing the rotational direction of the near-field for CCW and CW results in reduced or enhanced energy exchange and transverse diffraction, respectively. As a result, the CCW field produces a symmetric and narrow energy gain/loss spectrum, indicative of low phase matching and coherent interaction with a small diffraction angle. In contrast, the CW field causes a broader range of higher-order states, spanning within −300 eV < *E* < 300 eV, with distinct peaks at elevated azimuthal orders.

To compute the angular momentum distribution of the final electron wavepacket, a Fourier expansion in terms of azimuthal angular orders is employed: $\psi \left(x,y,z\right)=\sum_{m}\psi_{m}\left(\rho ,z\right)\;\exp\left(im\varphi \right)$ , where $\rho =\sqrt{x^{2}+y^{2}}$ is a radial component and *φ* is the azimuthal angle. Then, the angular momentum probability distribution [73], restricted to the azimuthal order, is calculated as $P_{m}=\int_{0}^{\rho }d\rho \;dz{\left|\psi_{m}\left(\rho ,z\right)\right|}^{2}$ to represent the azimuthal order distributions. However, we observe that the transformation of a single angular momentum order is not feasible; instead, the final electron wavepacket emerges as a complex superposition of multiple angular momentum orders (refer to Figure 4(e)–(h)). The CCW field applies a symmetric rotational force, analogous to a central potential, resulting in coherent lower-order angular momentum transfer. This behavior mirrors classical Rutherford scattering patterns observed in small-angle deflections. In contrast, the CW field generates an asymmetric, time-varying potential that scatters the electron wavepacket into higher-order angular momentum states, resembling high-energy scattering events with large-angle deflections. Similarly, panel (h) illustrates the azimuthal order distribution of the electron wavepacket under the influence of the *x*-polarized field. While linear polarization does not inherently carry angular momentum, a substantial angular momentum transfer is observed. This complexity arises because the *x*-polarized pulse creates a strongly oscillatory localized near-field that dynamically interacts with the electron wavepacket. The interplay between the electron motion and the non-uniform phase gradients in the near-field caused by plasmonic excitation and confinement generates these angular momentum distributions.

## 4 Conclusions

In conclusion, this study demonstrated the potential of plasmonic rotors as a powerful tool for manipulating free-electron wavepackets through controlled momentum transfer and energy modulation. By employing orthogonally polarized laser pulses with a phase offset, we excited circular dipolar near-fields in a gold nanorod and generated rotational plasmons with clockwise (CW) and counterclockwise (CCW) orientations. Our results reveal the intricate interplay between the direction of near-field rotation and the electron beam propagation, offering precise control over both linear and angular momentum exchange in the electron wavepacket. We showed that CW fields significantly enhance energy transfer and electron recoil due to stronger phase matching and increased coupling efficiency, whereas CCW fields exhibit narrower energy gain/loss distributions, indicative of reduced phase matching and weaker coupling. The unique characteristics of plasmonic rotors provide a versatile platform for advancing electron-beam shaping and harnessing coherent quantum interactions. These findings open new opportunities for integrating plasmonic rotors with other nanostructured materials to amplify coupling strength and expand the potential for high-resolution, low-energy electron microscopy. By extending these principles to more complex systems of rotational near-field excitations, this work lays a foundation for enhanced and active shaping of matter waves.
